# Reduction of D-dimer levels after therapeutic administration of antithrombin in acquired antithrombin deficiency of severe sepsis

**DOI:** 10.1186/cc3808

**Published:** 2005-09-19

**Authors:** Jordan Kountchev, Klaudija Bijuklic, Romuald Bellmann, Christian J Wiedermann, Michael Joannidis

**Affiliations:** 1 Resident, Medical Intensive Care Unit, Division of General Internal Medicine, Department of Internal Medicine, Medical University Innsbruck, Austria; 2 Resident, Laboratory for Inflammation Research, Division of General Internal Medicine, Department of Internal Medicine, Medical University Innsbruck, Austria; 3 Associate Professor, Medical Intensive Care Unit, Division of General Internal Medicine, Department of Internal Medicine, Medical University Innsbruck, Austria; 4 Professor, Division Head, 2nd Division of Internal Medicine, Department of Medicine, Central Hospital of Bolzano, Bozen, Italy; 5 Associate Professor, Director of the Medical Intensive Care Unit, Division of General Internal Medicine, Department of Internal Medicine, Medical University Innsbruck, Austria

## Abstract

**Introduction:**

In acute disseminated intravascular coagulation, the effect of antithrombin (AT) administration on elevated levels of D-dimer is not well established. In the present study, we report on changes in circulating levels of D-dimer in response to administration of AT in a series of patients with acquired AT deficiency due to severe sepsis.

**Methods:**

Eight consecutive critically ill medical patients presenting with acute disseminated intravascular coagulation associated with severe sepsis/septic shock received a single bolus infusion of AT over 30 minutes, aiming to achieve physiological AT levels. Haemostatic parameters including D-dimer were assessed prior to, 6 and 24 h after AT administration. An average of 42 ± 9 U/kg body weight was infused.

**Results:**

Following AT substitution, elevated levels of D-dimer fell whereas AT levels rose.

**Conclusion:**

These observations support the notion that AT can favourably affect fibrin degradation accompanying disseminated intravascular coagulation of severe sepsis.

## Introduction

Disseminated intravascular coagulation (DIC) is a systemic process potentially producing both thrombosis and hemorrhage. DIC is characterized by elevated levels of fibrin-related degradation products, prolonged prothrombin time (PT) and activated partial thromboplastin time (aPTT) as well as reduced levels of endogenous inhibitors of coagulation, such as protein C and antithrombin (AT). In the setting of severe sepsis, persistent high levels of fibrin(ogen) degradation represent a poor prognostic sign in patients with acute DIC [1]. On the other hand, marked reduction of AT levels at the onset of septic shock may be a sensitive marker of unfavorable prognosis, presumably by permitting persistence of the procoagulant state. Smaller phase I and II clinical studies recently demonstrated improvements in coagulation parameters after AT substitution (such as increased prothrombin activity as well as fibrinogen concentration), mediator levels and organ function [2]. A meta-analysis of randomized controlled trials showed significantly better survival rate with AT substitution [3].

To assure the effectiveness of AT in the treatment of patients with severe sepsis, the KYBERSept study, a multicenter, double-blind, placebo-controlled trial, was conducted, although it failed to provide definite and conclusive data on this issue [4]. Considering extensive preclinical evidence and the previously available data from several smaller clinical studies, it seems probable that it is not the lack of efficacy of AT that is responsible for the failure to reach the primary endpoint, but rather the study design, patient choice, dose and length of drug administration and also the endogenous pool of AT [5,6].

In acute DIC, little is known about the possible influence of AT substitution on elevated markers of hyperfibrinolysis, in particular D-dimer. We hypothesized that by means of its anticoagulant and anti-inflammatory actions, AT supplementation might reduce the coagulant factor consumption and hyperfibrinolysis accompanying DIC, which would be reflected by decreased levels of D-dimer. In the present study, we report on changes in levels of D-dimer in response to administration of AT in a series of patients with acquired AT deficiency due to severe sepsis.

## Materials and methods

### Patients

Eight consecutive critically ill medical patients presenting with acute DIC associated with severe sepsis/septic shock were analyzed during the period of January 2002 to December 2004. DIC was diagnosed according to the criteria defined by the International Society of Thrombosis and Haemostasis [7]. Study subjects represent all patients in whom AT was substituted for the treatment of acquired AT deficiency during the observed period, an indication currently approved in Austria. Underlying conditions were bacterial pneumonia (n = 3), pneumonia and myeloproliferative disorder (n = 2), spontaneous bacterial peritonitis with acute or chronic liver failure (n = 3). All patients had reduced AT levels. The mean Acute Physiology And Chronic Health Evaluation (APACHE) II score was 36 ± 2. Further patient characteristics are shown in Table 1. Two patients were receiving systemic heparin for anticoagulation during continuous renal replacement therapy (CRRT).

### Intervention

AT was from ZLB-Behring (Kybernin; Marburg, Germany). The dose was calculated and substituted as a bolus infusion over 30 minutes, aiming to achieve physiologic AT levels (AT 70% to 120% of normal). Haemostatic parameters including PT, aPTT, fibrinogen, antithrombin and D-dimer were measured four to six hours prior to and six hours after the AT substitution.

### Laboratory determinations

Haemostatic parameters were determined by routine laboratory methods. D-dimer was determined with a latex-enhanced turbidimetric test for the quantitative determination of cross-linked fibrin degradation products containing D-dimer in human plasma using a Dade Behring Coagulation Analyzer (Marburg, Germany). The test has a diagnostic sensitivity of 100% (95% CI 91–99.3) and specificity of 45.2% (95% CI 38.5–47.2). PT was determined coagulometrically using a Thromborel^® ^S reagent blood function analyzer system from Dade Behring. Platelet determination was done on an automated Sysmex NE 7000 hematology analyzer (Sysmex Corporation, Kobe, Japan).

### Statistical analysis

The SPSS 11.0 software package (Chicago, Illinois, USA) was used for statistical analysis. Results are given as mean ± SEM. Two-group comparisons were done using the Mann-Whitney U-test. Calculations of an association between D-dimer and AT levels were performed using the Spearman test. A p-value of <0.05 was considered significant.

## Results

In the majority of patients (six of eight), AT was used without concomitant heparin because it is known from the KyberSept study [4] that heparin might interfere with the actions of AT and increase the bleeding risk. A concomitant low-dose unfractioned heparin infusion was necessary, however, to keep the extracorporal circuit open in two patients requiring CRRT; the doses used were 400 IE/h for one patient and 600 IE/h for the other. The two patients receiving concomitant heparin were analyzed separately and were not included in the statistical analysis.

**Figure 1:**
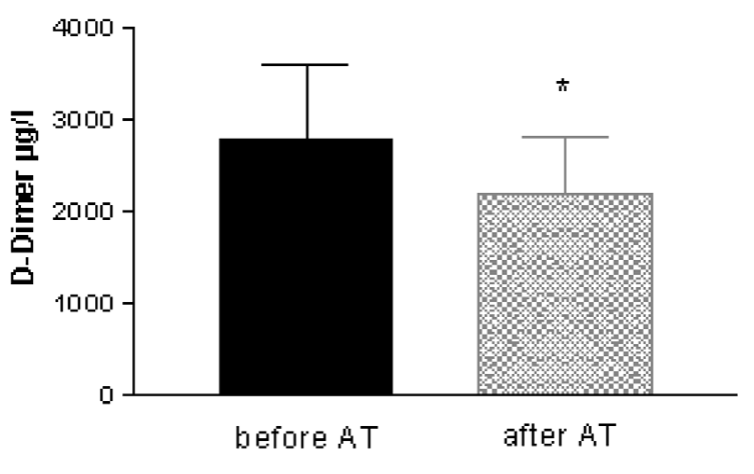
Effect of antithrombin (AT) substitution on D-dimer levels (results are given as mean ± SEM, n = 6; patients receiving low-dose unfractioned heparin not included; *p < 0.05).

**Figure 2:**
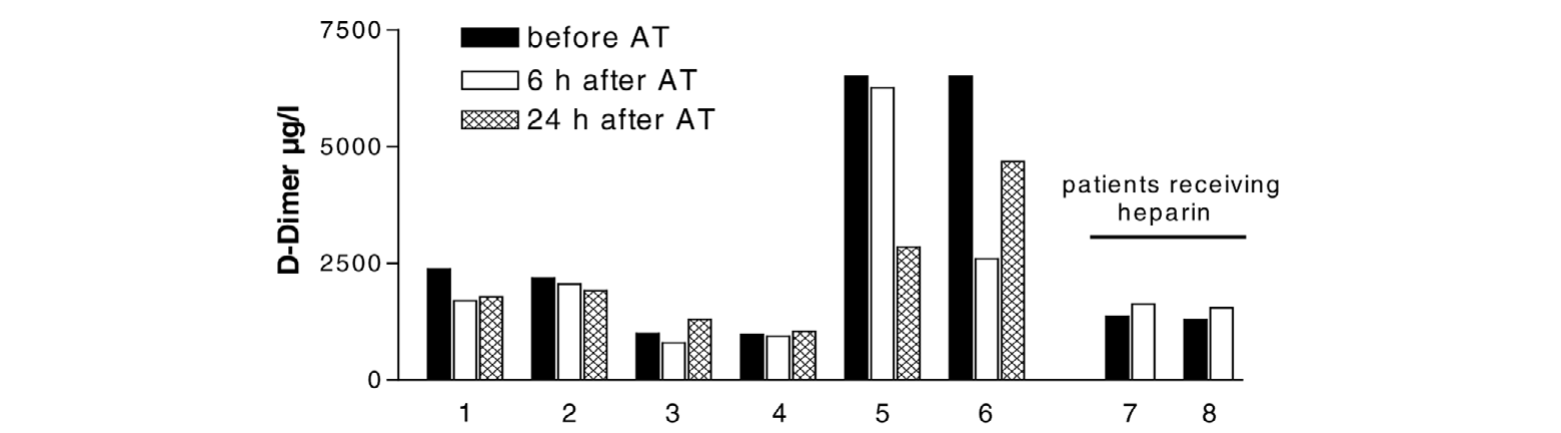
Effect of antithrombin (AT) on D-dimer levels in individual patients. D-dimer levels were assessed 4 to 6 h prior to, and 6 and 24 h after AT administration. Patients 7 and 8 were concomitantly receiving low-dose unfractioned heparin for continuous renal replacement therapy.

Haemostatic parameters before AT substitution were as follows: AT, 37 ± 7%; D-dimer, 3,254 ± 1,053 μg/l; PT, 45 ± 9%; and aPTT, 60 ± 5 s. Six hours after AT correction, laboratory parameters were re-determined. Administration of AT (mean doses administered were 2,500 ± 258 IE or 42 U/kg body weight) raised the AT levels from 37 ± 7% to 72 ± 5% (p = 0.027) of normal, as expected. Following AT substitution, a significant decrease in D-dimer levels from 3,254 ± 1,053 μg/l to 2,388 ± 2,014 μg/l (p = 0.028) was observed (Fig. 1 and 2), corresponding to a 26.7 ± 5.7% reduction of D-dimer levels. Interestingly, 24 h after AT supplementation, D-dimer levels increased again, but this increase did not reach statistical significance (Fig. 2). Administration of AT was not repeated according to local guidelines. None of the other parameters (aPTT, PT, fibrinogen, platelet count) showed any significant change. Notably, the two patients receiving heparin concomitantly to prolong circuit life during CRRT (400 and 600 IE per hour as a continuous pre-filter infusion of unfractioned heparin) demonstrated an increase in D-dimer levels (from 1,368 to 1,631 and from 1,291 to 1,544 μg/l, respectively, see also Fig. 2). No case of bleeding was observed in this small series of cases. An inverse relationship was observed between AT dose administered and the degree of D-dimer reduction (correlation coefficient r = -0.743; p = 0.09).

## Discussion

Fibrin-related degradation products, including D-dimers, play an integral role in diagnosing and monitoring acute DIC. D-dimer, which is derived from the degradation of cross-linked fibrin polymers, is a specific marker for increased procoagulatory activity, as well as fibrinolysis. Controlled and experimental observations in both humans and animals indicate that the elevation of fibrin(ogen) degradation products (FDP) and D-dimer is a constant feature of DIC, with marked elevations occurring rapidly after its initiation. Typically, the initial marked fibrinolytic response is dampened as (plasminogen activator inhibitor-1) PAI-1 levels rise, but D-dimer levels remain elevated [8]. Wada *et al*. [9] demonstrated that elevations in soluble fibrin, fibrin(ogen) degradation products, and D-dimer often precede overt DIC by several days. The D-dimer levels continue to rise thereafter as DIC progresses and subsequently declines with its clinical and laboratory improvement [9].

Elevated D-dimer levels *per se *have been shown to have a negative impact on survival in patients with DIC accompanying severe sepsis [1], presumably as a reflection of the ongoing and unopposed activation of coagulation and consumption of pro-coagulant factors. Furthermore, the optimal clinical effect and dose of activated protein C, presently the only hemostatically active substance showing benefit in the treatment of severe sepsis [10], although not representative for all patient groups and disease severity grades [11], was calibrated using changes in coagulation parameters such as D-dimer [12].

The literature addressing the effect of AT substitution on fibrin-related degradation products in the setting of acquired antithrombin deficiency of severe sepsis is rather scarce. Fourrier *et al*. [13] demonstrated a rapid decline in soluble fibrin levels after antithrombin administration. Soluble fibrin levels normalized within 24 h in 80% of the patients randomized to this therapy, whereas platelet counts showed little change until 5 days after the start of therapy. Uchiba *et al*. [14] used an endotoxin DIC model in rats and observed a 50% decline in fibrinogen at 6 h that was associated with an increase in serum fibrin degradation products of more than 100-fold. The decline in fibrinogen, increase in fibrin degradation products, and other manifestations of DIC were ameliorated by pre-treatment of rats with AT [14].

Carmassi *et al*. [15] described a significant reduction in plasmin-antiplasmin complexes, a marker of hyperfibrinolysis, after AT substitution in patients with liver cirrhosis and ascribed it to the improved antithrombotic potential of plasma after AT substitution; however, they did not measure D-dimer levels. Recently, Hoffmann *et al*. [16] showed a beneficial effect of long-term (14 days) high-dose (targeted AT activity >120%) AT substitution on coagulatory activation as well as effective reversal of coagulation abnormalities in patients with signs of DIC associated with severe sepsis in surgical critically ill patients. These effects were not primarily linked to a reduction of the fibrinolysis, as measured by plasminogen and α2 antiplasmin levels.

Our study, although small, is the first to show a significant decrease in D-dimer levels following AT substitution, indicating a possible acute early effect. All patients not receiving concomitant heparin showed consistent and significant reduction of D-dimer levels. Only the two patients receiving concomitant heparin as an anticoagulation regimen during CRRT failed to respond to AT. A possible explanation for this phenomenon may be that, although AT facilitates anticoagulation when applied systemically, its antithrombotic and anti-inflammatory actions may be inhibited in the presence of heparin at the cellular/endothelial level. Heparin is known to interfere with the action of AT by binding to the active site of the AT molecule, thus rendering it inert for interactions with endothelial glucoseaminoglycans and opposing the antithrombotic and anti-inflammatory actions of AT [17,18]. The interaction of AT with glucoseaminoglycans is thought to be responsible for the beneficial actions of AT. Intense concomitant administration of heparin may in fact have been an important reason for the overall negative results of the KYBERSept trial [4], which showed no significant reduction of mortality by day 28 in the setting of severe sepsis/septic shock. Actually, a *post hoc *analysis of the KYBERSept trial indicated that a subgroup of patients with sepsis and high risk of death (predicted mortality between 30% and 60%) treated with high-dose AT and not receiving concomitant heparin had significantly decreased mortality by days 28, 56 and 90 as compared to placebo [19].

As the influence of AT substitution on D-dimer levels has, to our knowledge, never been reported, this study represents the first attempt to reveal possible antithrombotic and maybe even antihyperfibrinolytic properties of AT in humans with DIC as indirectly monitored by the course of D-dimer levels. The consistent decrease of D-dimer levels associated with AT supplementation is supported by the fact that D-dimer levels increased again within one day following AT substitution. Despite the limited number of patients, the population studied reflects a broad spectrum of medical intensive care patients presenting with DIC.

Our study does have some potential pitfalls. The first concern is the rather limited number of patients, which may reduce the strength and general applicability of our data. As already mentioned, the studied patients represent the total population treated with AT for acquired AT deficiency of severe sepsis in our intensive care unit. Second, the patients underlying conditions that led to DIC are heterogenous and may *per se *cause hemostasiological abnormalities. On the other hand, systemic infection was present in every patient studied and was the direct cause for admission to the intensive care unit and the DIC was not previously presenting. Accordingly, the spectrum of diseases encountered represents the most common entities associated with DIC in clinical practice.

## Conclusion

Keeping in mind the small number of patients included in this study, we hypothesize that AT favorably influences consumption coagulopathy accompanying DIC as monitored by D-dimer levels. The decline in D-dimer levels after AT substitution may be indicative of a trend towards resolution of the procoagulant state seen in patients with sepsis and DIC. A prospective randomized study will be necessary to further elucidate this issue as well as reveal whether this will translate into diminished morbidity and mortality in patients with DIC accompanying severe sepsis and septic shock.

## Key messages

• Therapeutic substitution of antithrombin (AT) in patients with sepsis-associated DIC aiming at restoring normal al AT levels was associated with significant decrease of D-dimer levels.

• This effect of AT administration was not observed in patients concomitantly receiving heparin.

• D-dimer levels decrease observed may reflect inhibition of the procoagulant state associated with DIC in the setting of severe sepsis/septic shock.

## Abbreviations

aPTT = activated partial thromboplastin time; AT = antithrombin; CI = confidence interval; CRRT = continuous renal replacement therapy; DIC = disseminated intravascular coagulation; PT = prothrombin time; SEM = standard error of the mean.

## Competing interests

CJW received fees for speaking by Zlb-Behring, Baxter Vienna and Ely Lilly Austria.

## Authors' contributions

JK and MJ were responsible for the study concept, data acquisition, data interpretation and drafting of the manuscript. KB and RB were involved in the data acquisition, statistical analysis and in data presentation. CJW contributed to data interpretation and writing of the manuscript. All authors read and approved the final manuscript.

## Figures and Tables

**Table 1:** Patient characteristics and hemostatic variables before antithrombin substitution

Patient no.	Diagnosis	Organism	Age (years)	APACHE II score	PT (%)	aPTT (sec)	Fibrinogen (mg/dl)	AT (%)	D-dimer (μg/l)	Thrombocyte count	Outcome
1	Pneumonia, SBP	*Staphylococcus aureus*	56	38	30	73	263	20	2,374	47,000	Died
2	Pneumonia, SBP	*Staphylococcus aureus*	31	43	18	58	198	23	2,177	24,000	Died
3	SBP	No organism	56	39	37	51	266	39	976	38,000	Survived
4	Pneumonia	*Fusobacterium necrophorum*	19	35	71	56	490	36	999	55,000	Survived
5	Pneumonia	*Streptococcus pneumoniae*	50	28	37	77	290	34	6,500	89,000	Survived
6	Pneumonia, AML	*Klebsiella sp.*	61	30	45	45	80	53	6,500	87,000	Died
7^a^	Pneumonia, AML	*Escherichia coli*	62	32	60	50	690	34	1,368	11,000	Survived
8^a^	Pneumonia	*Staphylococcus epidermidis*	21	27	52	54	602	36	1,291	31,000	Survived

^a^Patients who received concomitant low-dose heparin. AT, antithrombin; aPTT, activated partial thromboplastin time; PT, prothrombin time. SBP = spontaneous bacterial peritonitis, AML = acute myelogenous leukemia
